# Draft Genome Sequence of a *Gordonia* sp. Isolated from the Soil of a Red Alder Plant

**DOI:** 10.1128/genomeA.01039-17

**Published:** 2017-09-28

**Authors:** Nicholas P. Devitt, Barrington Herman, Randi A. Luchterhand, Michael A. Costa, Jennifer L. Jacobi, Laurence B. Davin, Norman G. Lewis, Callum J. Bell

**Affiliations:** a National Center for Genome Resources (NCGR), Santa Fe, New Mexico, USA; b Institute of Biological Chemistry, Washington State University, Pullman, Washington, USA

## Abstract

A *Gordonia* species was cultured from soil of a red alder (*Alnus rubra*) plant. Here we present the assembled and annotated genome sequence to aid investigations into the potential of this organism as a symbiont and comparative studies of the genus *Gordonia*.

## GENOME ANNOUNCEMENT

The genus *Gordonia* is gaining attention due to its relevance in a variety of realms because its diverse metabolic activity has important implications for bioremediation; several *Gordonia* species degrade rubber, organic pollutants, and other xenobiotics. *Gordonia* species also have notable roles in agriculture and as opportunistic human pathogens (1). A filamentous microbe matching the description of a member of the phylogenetic order *Actinomycetales* was isolated from a red alder soil sample obtained in the Pacific Northwest. The isolate was cultured in modified (10%) Murashige and Skoog medium with full Gamborg’s vitamins. Genomic DNA was isolated with a GenElute bacterial genomic DNA kit (Sigma) following the protocol for Gram-positive bacteria used in conjunction with Lysozyme (Sigma).

The microbe sample was prepared for sequencing using the standard Pacific Biosciences 10-kb library prep protocol and sequenced on the PacBio RSII system. The resulting PacBio reads were assembled using the Falcon0.4 software (https://github.com/PacificBiosciences/FALCON). The assembly was further polished using PacBio’s quiver algorithm to increase genomic consensus accuracy (https://github.com/PacificBiosciences/GenomicConsensus). The assembly came together into 3 contigs covering 6,180,512 bp, with a maximum length of 5,136,039 bp and 67% GC content. To identify the assembled species, a BLAST search was done against NCBI’s nonredundant database, which showed the highest identity with *Gordonia polyisoprenivorans* (2). The contigs were then annotated using the NCBI Prokaryote Genome Annotation Pipeline (PGAP) (3). The PGAP annotation detected 5,530 coding sequences and 5,471 coding genes. The genome was also processed using PacBio’s BaseModification and motif detection pipeline in order to provide methylome data as a resource for future studies (see http://www.pacb.com/wp-content/uploads/2015/09/WP_Detecting_DNA_Base_Modifications_Using_SMRT_Sequencing.pdf) (4).

### Accession number(s).

This whole-genome shotgun project was deposited at DDBJ/ENA/GenBank under the accession number NQOE00000000. The version described in this paper is version NQOE01000000.
